# Validity of 12-Month Falls Recall in Community-Dwelling Older Women Participating in a Clinical Trial

**DOI:** 10.1155/2015/210527

**Published:** 2015-07-27

**Authors:** Kerrie M. Sanders, Amanda L. Stuart, David Scott, Mark A. Kotowicz, Geoff C. Nicholson

**Affiliations:** ^1^Department of Medicine, NorthWest Academic Centre, Western Health and the University of Melbourne, Sunshine Hospital, 176 Furlong Road, St Albans, VIC 3021, Australia; ^2^Research Institute of Health and Ageing, Australian Catholic University, 215 Spring Street, Melbourne, VIC 3000, Australia; ^3^School of Medicine and IMPACT Strategic Research Centre, Deakin University, P.O. Box 281, Geelong, VIC 3220, Australia; ^4^School of Clinical Sciences, Faculty of Medicine, Nursing and Health Sciences, Monash University, Monash Medical Centre, 246 Clayton Road, Clayton, VIC 3168, Australia; ^5^Department of Endocrinology and Diabetes, Barwon Health, Geelong Hospital, 1/75 Bellerine Street, Geelong, VIC 3220, Australia; ^6^Rural Clinical School, The University of Queensland, 152 West Street, Toowoomba, QLD 4350, Australia

## Abstract

*Objectives*. To compare 12-month falls recall with falls reported prospectively on daily falls calendars in a clinical trial of women aged ≥70 years. *Methods*. 2,096 community-dwelling women at high risk of falls and/or fracture completed a daily falls calendar and standardised interviews when falls were recorded, for 12 months. Data were compared to a 12-month falls recall question that categorised falls status as “no falls,” “a few times,” “several,” and “regular” falls. *Results*. 898 (43%) participants reported a fall on daily falls calendars of whom 692 (77%) recalled fall(s) at 12 months. Participants who did not recall a fall were older (median 79.3 years versus 77.8 years, *P* = 0.028). Smaller proportions of fallers who sustained an injury or accessed health care failed to recall a fall (all *P* < 0.04). Among participants who recalled “no fall,” 85% reported zero falls on daily calendars. Few women selected falls categories of “several times” or “regular” (4.1% and 0.4%, resp.) and the sensitivity of these categories was low (30% to 33%). Simply categorising participants into fallers or nonfallers had 77% sensitivity and 94% specificity. *Conclusion*. For studies where intensive ascertainment of falls is not feasible, 12-month falls recall questions with fewer responses may be an acceptable alternative.

## 1. Introduction

The major clinical outcome of osteoporosis is an increased risk of fragility fractures [1]. Approximately 90% of hip, forearm, and pelvis fractures result from a fall [2], and so falls monitoring is important in clinical practice and research settings. The incidence of falls among community-dwelling older adults varies widely between studies [3] but it is generally reported that between 30% and 60% fall each year [4]. The wide range is attributable to not only differences in the study populations and definitions of a “fall” but also the methods of falls ascertainment. When ascertained by recall, the interval of recall is obviously important; one 12-month study reported a more than threefold variation in fall rates among older men when falls were ascertained by varying intervals of recall. Men asked to recall falls monthly had a fall rate of 21% compared with 16% for those asked 3-monthly and 6% for those asked 12-monthly. For females in this study the rates were 26%, 18%, and 21%, respectively [5]. Fujimoto and colleagues conclude that the difference in falls rate may be due to differences in the method of recollection since the participants were matched for falls risk factors. Furthermore, accuracy of 12-month falls recall decreases from almost 80% in older adults who do not fall to 20% in older adults who have fallen on three or more occasions during that period [6]. Thus, where accurate data on all falls is crucial to the study outcomes, it is recommended that falls information be collected at weekly or monthly intervals [7]. Nevertheless, for many studies falls information is not the primary outcome and such labour intensive ascertainment is not practical. In such studies participants are often asked to recall the number of falls in the past 12 months, yet the accuracy of this in older people is often questioned. There is little information available from studies performing a head-to-head comparison of prospective daily falls reporting and 12-month falls recall in older adults.

## 2. Aim

The aim of this study was to compare 12-month falls recall with falls reported prospectively on daily falls calendars in a clinical trial of women aged ≥70 years.

## 3. Materials and Methods

This analysis is part of the Vital D study—a randomised, placebo-controlled double blind trial investigating whether a large annual dose of cholecalciferol (vitamin D) reduces falls and fractures in older women. As part of this study, fall events were intensively monitored over the entire intervention period of three to five years (2003–2008) [8]. Using a questionnaire, participants were asked during 2007 to select the category that best described their frequency of falls in the last 12 months. This 12-month recall of falls was compared with results from our database. The database represents a record of falls ascertained each month from a daily calendar for all 2,096 participants, as detailed below. Falls recorded on daily calendars were followed up with a standardised questionnaire administered by telephone regarding the characteristics and consequences of the fall. All participants had completed monthly falls calendar for at least two years before completing the 12-month recall of falls ascertainment. The method of falls ascertainment by prospective calendar returns is referred to as “daily falls calendar” and “12-month recall of falls” refers to results from categorical responses from a question regarding recall of falls in the past 12 months.

### 3.1. Participants

Between June 2003 and June 2005 we recruited 2,317 older women who were at high risk of falls and fragility fracture. To be eligible, women needed to be at least 70 years of age and not residing in a supported residential aged care facility. All participants scored at least 5 points on a tool based on risk factors for hip fracture identified by Cummings and colleagues [9] including a history of any fracture since the age of 50 years, maternal history of hip fracture, current body weight less than 50 kg, falling in the past year, poor vision (1 point each), and age 80 years or more (2 points).

### 3.2. Fall Definition and Recording

A fall was defined as “an event reported either by the faller or by a witness, resulting in a person inadvertently coming to rest on the ground or another lower level, with or without loss of consciousness or injury” [10]. This definition was included on our study newsletter sent to all participants twice a year.

### 3.3. Falls Ascertainment

#### 3.3.1. Daily Falls Calendar

On enrolment into the study participants were given a 15-month falls calendar comprising a set of monthly postcards. The calendars were renewed postannually with a three-month overlap to allow for delays in the mail or other contingencies when the calendar was due for renewal. Participants completed falls calendars daily by writing “*F*” if they had a fall, fracture, or both or “*N*” if not. Calendars were backed with magnetic strips to enable attachment to refrigerators, where they would be seen frequently. Each postcard included the participants' unique study number, the address of the study centre, and prepaid postage for monthly returns. Participants who had not returned their postcard within two weeks of the end of the month were telephoned and asked about falls in the previous month. When a fall or fracture was recorded, a standardised questionnaire was administered by telephone, and fractures were radiographically confirmed.

#### 3.3.2. 12-Month Falls Recall

During 2007 all participants were sent a study questionnaire of eight questions relating to falls, past history of fracture, and sun exposure habits during summertime. The falls recall question was set out as follows.

*Have you had any falls in the last twelve months?*

*Never  [](0)*

*A few times  [](1)*

*Several times  [](2)*

*Regularly  [](3)*

Participants who did not return the questionnaire were interviewed over the phone.

### 3.4. Statistics

To compare the two methods of ascertainment, the continuous falls data from daily falls calendars were categorised into four responses (never; a few times; several times; and regularly) from the recall question using the best agreement between the sum of self-reported daily falls and the 12-month falls recall response.* Sensitivity* was defined as the number of women whose 12-month falls recall response matched the category they were allocated to according to daily falls calendar (a few times; several times; and regularly), divided by the total number of women in that falls category.* Specificity* was defined as the number of women whose 12-month falls recall response was “never” and whose daily falls calendar total was zero, divided by the total number of women who did not report a fall using the daily falls calendar. The* negative predictive value* was defined as the proportion of participants who were “nonfallers” according to both the daily falls calendar and the 12-month recall (true nonfallers), divided by all participants who selected “no fall” on the daily fall calendar regardless of their response on the 12-month falls recall questionnaire (true and false nonfallers). The likelihood ratio was calculated to estimate the probability/likelihood that a “several or regular falls” response from the 12-month falls recall questionnaire was the same as the categorisation from daily falls calendar (sensitivity/(1 − specificity)). McNemar's test compared the proportion of fallers (determined by daily falls calendars) who did not report a fall in the 12-month falls recall response, according to radiographically confirmed fracture, and self-report of visiting a doctor, hospitalisation, or injury, ascertained from daily falls calendar follow-up interviews.

All statistical analyses were performed using Minitab (version 13) except for McNemar's tests which were performed in SPSS (version 22).

## 4. Results

The analysis includes 2,096 participants with complete daily falls calendar data and 12-month falls recall data for the same 12-month period. During the twelve months, 43% (*n* = 898/2096) of participants reported a fall according to the daily falls calendar. Of these, 77% (*n* = 692/898) recalled having at least one fall according to the 12-month falls recall question (sensitivity 77%). Of the fallers, 40% (*n* = 360/898) had more than one fall (Table 1). Fallers were slightly older than the nonfallers (median (IQR) fallers versus nonfallers: 78.9 years (75.5 to 82.8 years) versus 77.7 years (75.0 to 81.1 years), *P* < 0.001) and were over three times more likely to have had a fall in the year prior to this 12-month recall interval (odds ratio (95%  CI) age-adjusted: 3.21 (2.68; 3.85), *P* < 0.001).

From the daily falls calendar, 26% of participants had one fall, 10% had two falls, and only 7% had more than two falls. The 12-month falls recall data does not allow us to distinguish one and two falls but 32% of all participants recalled falling “a few times” and 4.5% recalled falling “several times” or “regularly” (Table 1). The group of 206 participants who reported a fall in daily falls calendars but did not recall falling in the 12-month falls recall (23%, *n* = 206/898) was older than others who recalled the same response on both falls ascertainment methods (median 79.3 years versus 77.8 years, *P* = 0.028). Only 6% of participants who did not record a fall on daily falls calendars incorrectly recalled a fall in the past 12 months (94% specificity; *n* = 1128/1198) and 85% of women who selected “no fall” on the 12-month falls recall were correct (negative predictive value 0.85; *n* = 1128/1334) (Table 2). Of the 70 women who did not report a fall on daily falls calendars but incorrectly selected that they had fallen on the 12-month falls recall (6%; *n* = 70/1198), only 36% (*n* = 25/70) had a fall in the three months prior to the 12-month recall period. Of the 206 participants who did not recall a fall on the 12-month question, 70% reported falls on daily calendars that occurred in the first six-month period of ascertainment. When participants were classified into just two categories of “fallers” or “nonfallers” there was good agreement between the daily falls calendar and 12-month falls recall data (Kappa 0.73, 95% CI: 0.68, 0.79).

The best agreement between the daily falls calendar and the 12-month falls recall questionnaire was achieved by defining “a few times” as one to four falls and “several” as five to seven falls per year (Table 3). The sensitivity of the higher fall categories was low regardless of the number of falls used to define the categories (30% to 36%; Tables 1 and 2). Using our results to calculate sample size [11] we estimate studies need to have at least 2,000 person-years to have the power to detect a difference between the two higher fall categories of “several” and “regular” fallers (80% power and 0.05 significance level). This is based on recruitment of a similar “at risk” cohort. By comparison a total sample size of 400 to 430 person-years would be needed to detect a difference between “fallers” and “nonfallers.” This sample size should also have sufficient power to detect a difference between fallers of “a few times” versus “several/regular” fallers since the difference in the proportion of participants classified as “few times” (41%) versus “several/regular” (1.5%) is larger than the difference between fallers (43%) and nonfallers (57%). The likelihood ratio for the two combined categories of several or regular falls is 7.5 (sensitivity/(1 − specitivity): 0.3/(1 − 0.96)). Thus, a woman who recalls falling several times or regularly is 7.5 times more likely to be correct rather than incorrect in her selected falls frequency category.

Among the 898 women who reported one or more falls on daily falls calendars during the study period, 80 (9%) had a radiographically confirmed fracture. There were a total of 1605 falls, 1566 (98%) of which were able to be investigated further by telephone interviews. 414 (26%) and 70 (5%) falls incidents resulted in a doctor visit or hospitalisation, respectively. A total of 705 (79%) participants reported sustaining an injury, 321 (36%) reported visiting a doctor, and 70 (8%) reported being hospitalised, on at least one occasion due to a fall. As reported in Table 4, amongst participants who reported a fall on daily falls calendars, significantly smaller proportions of women who had a radiographically confirmed fracture, or who reported being hospitalised, seeing a doctor, or sustaining any injury, recorded no falls in response to the 12-month falls recall question.

## 5. Discussion

In our cohort of over 2,000 older women selected on the basis of being at higher risk of falls or fractures, over half (57%) did not prospectively report a fall over a 12-month interval, approximately one-quarter (26%) reported one fall, and fewer than one in five (17%) reported falling twice or more. Fallers were 3.2 times more likely to have fallen in the previous year (prior to the study period) than nonfallers and were slightly older than nonfallers. This proportion of fallers (43%) is consistent with other estimates of 40% for older women [12] and estimates of 10% having two or more falls [13], since the incidence of falls increases with age and our cohort was older and was specifically recruited to be at higher risk of falls and fracture.

The head-to-head comparison of ascertainment methods shows, in response to a 12-month falls recall questionnaire, that 82% (*n* = 1727/2096) of participants matched prospectively reported daily falls totals. However nonfallers and those that fell only a few times were more likely to match these responses (94% identical for nonfallers and 68% identical for “a few times” fallers). Few participants reported falling more than four times in one year in daily falls calendars (1.5%; *n* = 32/2096) and, of those, half did not recall having several or regular falls in response to the 12-month falls recall question (*n* = 16/32), which is consistent with previous Australian data indicating poor 12-month falls recall in older adults who fall three or more times in a year [6]. Although there were 70 (6%) women who reported no falls on daily calendars but reported falling on the 12-month falls recall, the general classification of fallers or nonfallers has a high sensitivity and specificity (77% and 94%, resp.). As participants in the Vital D study had been completing the monthly falls calendar for several years, we were able to ascertain that only 36% of the 70 women who reported a fall on the 12-month recall but not daily falls calendars had sustained a fall in the 3 months prior to the specific 12-month recall interval.

Twenty-three percent of participants who reported a fall on daily falls calendars in our cohort did not recall falling in the past year (*n* = 206/898). This is consistent with proportions reported by Cummings and colleagues (13% to 32%) but is almost double the 13% reported for their 12-month recall group [14]. There are substantial differences between the two cohorts. Participants in the study by Cummings were 304 men and women aged 60 years and older, whereas our cohort of women only was almost seven times larger and aged at least 70 years (37% of Cummings cohort aged <70 years). Furthermore, our participants who failed to recall fall(s) were slightly older than those who correctly recalled a fall (79.3 versus 78.1 years, *P* = 0.028).

It has been suggested that participants who fail to recall falling may have difficulty placing the fall in time and that asking whether a fall had occurred since some other dated event that the person remembers may improve the accuracy of recall [14]. This may have contributed to the substantial disparity in the proportion of participants that forgot a fall (current study versus Cumming: 23% versus 13%, resp.) since our recall period did not coincide with the commencement of the study (an “event” often remembered by participants). Nevertheless only 18% (*n* = 34/206) of those who reported a fall on daily falls calendars but not 12-month falls recall questionnaire reported a fall in the three months prior to the study period although 58% (*n* = 119/206) reported a fall in the year prior to the recall interval. Similarly only 36% of nonfallers (according to daily falls calendars) who reported falling on the 12-month falls recall questionnaire had sustained a fall in the three months prior to the recall interval. Our sensitivity (77%) was similar to the 6-month recall (74%) but lower than the 12-month recall (87%) reported by Cummings et al. [14], and this may be related to the older age of our cohort which may be associated with poorer recall. Specificity of both studies was similar (current study and Cummings et al.: 94% and 93%, resp.).

We also observed that fallers (according to daily falls calendars) were around twice as likely to not report a fall in response to the 12-month falls recall question, if they did not sustain a radiographically confirmed fracture or report any injury or health care utilisation at the time of the fall. This is consistent with a previous study of 12-month falls recall in Australian older adults that reported 87% and 62% recall accuracy for injurious and noninjurious falls, respectively [6]. Thus, it is likely that falls rates are underestimated when falls do not result in injury or health care utilisation.

In this study one-quarter of all falls incidents resulted in a GP visit and, for 5% of falls incidents, hospitalisation. This is consistent with previous estimates indicating that approximately 20% of all fall incidents require medical attention [15]. Approximately 15% of all participants reported accessing a GP because of a fall incident in this study but this is significantly higher than recent estimates from the Belgian older adult population indicating that approximately 2.5% of noninstitutionalised general practice patients received GP care for fall-related injuries [16]. The difference is likely attributable to our recruitment of older women identified as having increased risk of falls or fractures who would therefore be more likely to access health care for fall-related injuries than the general older adult population. Conversely, only 8% of our participants reported being hospitalised due to a fall, compared to 31% in the previous study. This may be explained by the similar differences in falls-related fractures; 9% of participants in our study, compared to 32% of participants in the Belgian study [16], sustained a fracture. The higher fracture and hospitalisation rates in the previous study are probably reflective of the fact that the study populations were older adults accessing GP care, indicating that the fall-related injuries captured were in the most severe range (e.g., fracture) and more likely to result in hospitalisation.

A limitation of this study is that half the participants were randomised to high-dose vitamin D supplementation, which was observed to increase risk of falls and fracture in this population [8]. In a post hoc analysis, we observed that, amongst women who were fallers according to daily falls calendars, a smaller proportion of those receiving vitamin D supplementation classified themselves as nonfaller in response to the 12-month falls recall question, compared to those receiving placebo (20 versus 26%; *P* = 0.048). The improved 12-month recall of the vitamin D group may be explained by the higher rate of falls in this group or could be related to an effect of vitamin D, with a recent systematic review indicating that higher vitamin D status is associated with improved cognitive function in older adults [17].

The head-to-head comparison of the falls data also poses an unavoidable limitation of the study since the participants recalling their falls over the past 12 months had also been posting a daily record of their falls each month over the same period. The 23% of participants who reported falls using prospective daily falls calendars but did not report a fall in response to the 12-month falls recall question are likely to be an underestimation of prevalence of forgotten falls, as 12-month recall of falls may be improved by the daily calendar ascertainment in this study. Furthermore, recall of the falls event may have been reinforced by the follow-up telephone interviews. Even with the most rigorous reporting methodology, it is quite likely that falls are underreported [12]. We and others [13] have noted that denial can be a factor in underreporting as some older people take pride in being a “nonfaller” and “do not want to blot their copybook” by reporting a fall. Older people can blame external factors for their fall and not count it as a “true” fall. Overreporting using the daily falls calendar is unlikely since recorded falls were confirmed by telephone and the circumstances of the event were recorded on our database.

Many cohort studies relying on recall of falls over a 12-month period are unlikely to be adequately powered to show differences between groups in the higher falls categories since few participants accurately self-select higher fall frequencies (sensitivities ~30%). Sample size calculations suggest that studies with 2,000 person-years may be powered to detect a difference between fallers of “several times” versus “regular fallers.” Studies with 400 person-years may be sufficiently powered to detect a difference between fallers and nonfallers and also between fallers of “a few times” and more frequent fallers. Although we were unable to calculate the reliability of older women selecting between “one fall” and “more than one fall” for the 12-month falls recall question, a choice of three categories is likely to provide a better “spread” of the data since our cohort of over 2,000 older women had 57% nonfallers, 26% with one fall, and 17% with two or more falls. These three categories might offer more insight into risk factor associations than the simple classification of fallers/nonfallers. Nevertheless when the four categories of falls recall were combined, the classification of fallers/nonfallers captured 77% of fallers and correctly identified 94% of nonfallers and may provide a reasonable alternative for smaller studies where falls are not the primary outcome and an intense ascertainment of fallers is not feasible.

## 6. Conclusions

With the “ageing” of most western populations, the consequences of injurious falls and their impact on both quality of life and the economic burden to the health system continue to grow. We hope, by reporting this head-to-head comparison of prospective daily falls calendars and 12-month falls recall, that researchers can make informed choices in designing studies that incorporate some falls risk data in which a more intensive ascertainment of fall events is not feasible.

## Figures and Tables

**Table 1:** Proportion of participants by number of falls.

Daily falls calendar		12-month falls recall question	
Number of falls reported	Proportion of participants (*N*)	Category selected	Proportion of participants (*N*)
None	57.2% (1198)	Never	63.4% (1334)
1	25.7% (538)	A few times	31.8% (667)
2	9.8% (205)	Several times	4.1% (86)
3	3.8% (80)	Regularly	0.4% (9)
4	2.1% (43)		
5	0.6% (13)		
6	0.4% (8)		
>6	0.5% (11)		

Total	100% (2096)		100% (2096)

**Table 2:** Table of frequencies: fallers and nonfallers.

12-month falls recall	Daily calendar		
	Falls, *N* (%)	No falls, *N* (%)	
Falls	692 (77%)	70 (6%)	762
No falls	206 (23%)	1128 (94%)	1334

	898	1198	2096

**Table 3:** Cross tabulation of falls by best agreement between monthly ascertainment and 12-month recall category question.

12-month falls recall	Daily falls calendar				Sensitivity of 12-month recall
	No falls	Few (1 to 4 falls)	Several (5 to 7 falls)	Regularly (8+ falls)	
No falls	**1128**	203	3	0	
Few	65	**589**	11	2	68%
Several	5	70^1^	**7**	4	30%
Regularly	0	4	2	**3**	33%

	1198	866	23	9	

^1^Only 14% (*n* = 10/70) had 4 falls, so reclassifying the criteria of the “few” category to be only 1 to 3 falls did not improve the agreement between the daily falls calendar totals and the 12-month falls recall. Sixty-five percent of these women fell only once or twice (n = 46/70).

**Table 4:** 12-month falls recall according to injury or health care utilisation.

12-month falls recall	Confirmed fracture		Fall interview					
	Yes	No	Saw a doctor		Hospitalised		Any injury	
			Yes	No	Yes	No	Yes	No
No falls, *N* (%)	8 (10)	198 (24)	45 (14)	161 (28)	9 (13)	197 (24)	133 (19)	73 (38)

*P* value^*∗*^	**<0.001**		**<0.001**		**<0.001**		**<0.001**	

^*∗*^McNemar's test.
